# Clinical Reflections

**Published:** 1883-08

**Authors:** Ad. Lippe

**Affiliations:** Philadelphia


### CLINICAL REFLECTIONS.

Ad. Lippe, M. D., Philadelphia.

On the morning of the 7th of June a lady called about 10 A. M., asking for some medicine for her brother, who for more than two days had suffered from a severe nose-bleeding and was not able to arrest it. Not able to learn more than this from her, I gave her a few doses of Cactus grandiflorus, and learned that the first dose had arrested the bleeding, but that it had returned again, was better during the night, but worse on the 8th. On the evening of that day I finally obtained a full history of the case. The gentleman, fifty years old, had always enjoyed robust health—in fact, was a picture of health; he stood six feet in his stockings, was well nourished, being a rather high liver. His father had been a still larger man, and had died of apoplexy at the age of sixty-five years. On the 5th of June a complete hemorrhage from the left nostril had set in; his family physician, an allopath and a learned professor in one of our celebrated medical colleges, applied alum and plugged up the nostril—of course to no purpose. In the night of the 6th the hemorrhage became worse, and during the night another learned man was called in in consultation; and now the patient had to take first a bottle of Citrate of Magnesia, which was followed up by liberal doses of the tincture of Secale Cornutum; but the bleeding did not stop, and on the 7th day of June, the friends becoming very much alarmed, almost as much so as the patient, resolved to seek other help; but not at first knowing anything more of the case than what his sister reported, could not well understand why he continued to have more attacks of nose-bleed. Drugged, having suffered besides all day of the 7th with incessant debilitating diarrhœa, utter loss of appetite, and rapidly becoming weaker. I finally learned further that he was free from nose-bleed now when in a recumbent position, but worse when he arose *or when he stooped.* When he arose his head felt full, he felt faint, and if he stooped the blood poured out in a stream. A single dose of Rhus tox.$^{cm}$ (Fk.), which I gave him on the evening (9 P. M.) of the 8th of June, caused him to sleep all night till 8 A. M. He then arose, as he expressed himself, to feel like a new man, had a good breakfast, and reported himself, 10 A. M., at the office well and disgusted with the allopaths.

*Comments.*—An apparently simple case of epistaxis became unmanageable in the hands of our scientific opponents. Such an apparently simple case, becoming unmanageable, calls us "to reflect." The old school—old to be sure, but utterly void of any guiding principles, blind to the simplest truths, thoughtless as to a correct diagnosis to guide it in therapeutics—this aged school could not even manage a case of epistaxis. What, then, will the close observers ask—what, then, do the followers of that common school cure? If they sometimes accidentally restore the sick, if the sick do not die outright, they never *cure* or restore the sick to better health than he enjoyed before he sickened. All the accidental recoveries under the allopathic treatment are made under the law of the Similars, as Hahnemann so clearly demonstrated. It is a prevailing notion that the pathology of to-day is far superior to the pathology of Hahnemann's days, and should receive better acknowledgment from the members of the homœopathic school. It has been charged that Hahnemann rejected pathology altogether, which is a fatal error; he did reject it as a basis of therapeutics, and whatever advances have been made in this collateral branch of medical science will be utilized by every progressive healer. Pathology will enable us to obtain the symptoms of the sick, and will continue to teach us the respective value of these symptoms; will enable us to discern more accurately the most important symptoms—that is, the symptoms belonging to the individual and not necessarily to the disease, pathologically speaking, from which he is suffering. It will enable us to judge of the new symptoms arising in a case of sickness, whether they indicate the progress of the disease or whether they belong to symptoms denoting a salutary crisis. The case above related shows that the most progressive scientific men of the common school of medicine do not appreciate the knowledge imparted to them by pathology as it stands to-day. The old school forever demanded, as a ruling axiom, "*Tolle causam?*" Now, in this case, the cause of the disorder was evidently congestion of blood to the head, and was it rational treatment to apply means by which the capillary vessels were to be first contracted by Alum, later by Secale Cornutum, or was the congestion to the head to be relieved by a cathartic?

If that is a specimen of rational allopathic treatment, we may well feel deep pity for the victims who confide in such irrational and unscientific treatment. We may ask, what would a homœopathician gain if the "New Code" was introduced? Is it desirable to consult with that sort of scientific men? The true healer will continue to accept the totality of symptoms as the only guide in his therapeutics. In this case we have a knowledge of many remedies causing nose-bleed, and in this, as in all other cases, individualization must guide us in the selection of the curative remedy, if we find the similar among the many proved drugs. In this case the most striking symptom was an aggravation from stooping, which is characteristic of Rhus tox. Again, the strong aggravation of the head symptoms when rising from a recumbent position is also characteristic of Rhus tox. Again does true pathology teach us that a nose-bleed frequently relieves the congestions to the head in diseases, as in typhus fever, that, therefore, the mere local contraction of the capillaries is not a rational remedy. Rhus tox. being found the true similar remedy, was administered in such dose as a long experience has taught us to be most effective in the very large majority of cases.

Ridiculous as it is, the attempt has been made to determine at what grade of potencies the curative power ceases. According to the notion of these proponents, we are to be confined to the sixth, tenth, twelfth, or, at furthest, to the fifteenth potency. Even these men, only anxious for "recognition," do sadly disagree, and well they may. This is the one extreme; the other extreme is still more ridiculous. We find the inventor of a new law of cure, by him "discovered," invite the homœopathic healer, and more especially those who habitually use high potencies, to "progress" and accept the new law that the product of a disease, "*if highly potentized,*" will cure the disease itself. Returning to the case related, we should be much edified if this discoverer and manufacturer of highly potentized products of a disease would inform us how we could well successfully have applied his newly discovered law. We take it for granted that said discoverer is a scientific physician, and is fully posted in pathology; he must, therefore, be fully aware that there exist active and passive epistaxis, idiopathic or symptomatic, from both or either nostril, or it changes into chaonorrhagia; he undoubtedly knows that in affections of the liver the right nostril generally bleeds, while in affections of the spleen the left nostril bleeds generally; he also must know that there exist a great variety of causes for epistaxis, in putrid fevers, mechanical injuries, congestion to the head, polypus of the nose, suppressed menstruation, etc. The causes have surely to be fully considered. If, then, epistaxis is to be considered the product of the disease, this product, so differently discharged, having such differing causes, would, each specimen of it, have to be highly potentized by itself. Surely this learned discoverer could not expect any conscientious physician to administer the highly potentized blood taken from a woman suffering from suppressed menstruation, to cure the nose-bleed of a person who had received a knock on the nose. Absurd! we are not yet ready to become the laughing-stock of rational medical men of any school.

The question of the dose remains an open question, and the time for even earnestly discussing it has not come yet. Let us first settle the first question, the question whether, as homœopathists, we are not bound to acknowledge the law of the similars as the only true, eternally true, law of cure. The man who advocates isopathy is surely imbued with the fallacious notion that the law of the similars can be set aside and the law of the idem be accepted; that the proving of drugs is to be set aside and that the product of a hypothetical disease is so changed by potentization that that process makes it homœopathically indicated for the cure of hypothetical disease. The homœopathic law of cure is applicable under all circumstances; the law of *æqualia æqualibus* is no law at all, as it is *never* truly applicable. Will the learned discoverer let the homœopathists know how he will cure the nose-bleed under *his law?* or will he confess that as far as he knows Homœopathy is successful, far more successful, than any other school of medicine in curing the sick? And furthermore, it would be a charitable act of this learned discoverer if he would give some plausible reason for administering products of disease, when we find in every volume of the old writers mention made of the curative powers of innumerable drugs, now forgotten and set aside, as the Bee Poison was, because these occasionally beneficial drugs had been paraded before the medical world as "specifics," just as the discoverer of the new law parades his manufactured highly potentized products of disease as "specifics." Every thinking healer knows that there do not exist specifics for diseases, and that they never will be found. The true and only possible progress forward in the healing art can be attained if we prove drugs formerly used and now forgotten. We will then do what Cullen caused Hahnemann to do—find out *under what circumstances* medicinal substances like Cinchona will cure diseased conditions. The known provings of Rhus tox. enabled us to *cure* the above case under the law of cure belonging exclusively to our school. Nobody desires a new law just now.

